# Parthenogenetic mosaicism: generation via second polar body retention and unmasking of a likely causative *PER2* variant for hypersomnia

**DOI:** 10.1186/s13148-021-01062-0

**Published:** 2021-04-07

**Authors:** Yohei Masunaga, Masayo Kagami, Fumiko Kato, Takeshi Usui, Takako Yonemoto, Kazuo Mishima, Maki Fukami, Kazushi Aoto, Hirotomo Saitsu, Tsutomu Ogata

**Affiliations:** 1 Department of Pediatrics, Hamamatsu University School of Medicine, Hamamatsu, Japan; 2 Department of Molecular Endocrinology, National Research Institute for Child Health and Development, Tokyo, Japan; 3 Department of Medical Genetics, Shizuoka General Hospital, Shizuoka, Japan; 4 Department of Diabetes and Endocrinology, Shizuoka General Hospital, Shizuoka, Japan; 5 Department of Psychiatry Section of Neuro and Locomoter Science, Akita University School of Medicine, Akita, Japan; 6 Department of Biochemistry, Hamamatsu University School of Medicine, Hamamatsu, Japan; 7 Administration Department, Hamamatsu Medical Center, Hamamatsu, Japan

**Keywords:** Parthenogenesis, Second polar body retention, Idiopathic hypersomnia, *PER2*, Unmasked variant

## Abstract

**Background:**

Parthenogenetic mosaicism is an extremely rare condition identified only in five subjects to date. The previous studies indicate that this condition is mediated by parthenogenetic activation and is free from a specific phenotype ascribed to unmaking of a maternally inherited recessive variant in the parthenogenetic cell lineage.

**Results:**

We examined a 28-year-old Japanese 46,XX female with Silver-Russell syndrome and idiopathic hypersomnia. The results revealed (1) predominance of maternally derived alleles for all the differentially methylated regions examined; (2) no disease-related copy-number variant; (3) two types of regions for all chromosomes, i.e., four BAF (B-allele frequency) band regions with single major microsatellite peaks of maternal origin and single minor microsatellite peaks of non-maternal (paternal) origin, and six BAF band regions with single major microsatellite peaks of maternal origin and two minor microsatellite peaks of maternal and non-maternal (paternal) origin; (4) an unmasked extremely rare *PER2* variant (c.1403G>A:p.(Arg468Gln)) with high predicted pathogenicity; (5) mildly affected local structure with altered hydrogen bonds of the p.Arg468Gln-PER2 protein; and (6) nucleus-dominant subcellular distribution of the p.Arg468Gln-PER2 protein.

**Conclusions:**

The above findings imply that the second polar body retention occurred around fertilization, resulting in the generation of the parthenogenetic cell lineage by endoreplication of a female pronucleus and the normal cell lineage by fusion of male and female pronuclei, and that the homozygous *PER2* variant in the parthenogenetic cells is the likely causative factor for idiopathic hypersomnia.

**Supplementary Information:**

The online version contains supplementary material available at 10.1186/s13148-021-01062-0.

## Background

Normal mammalian development requires both maternal and paternal genomes, because of the presence of multiple imprinted genes [1]. Thus, parthenogenetic mammals are incompatible with life because of absent expression of paternally expressed genes and excessive expression of maternally expressed genes, and androgenetic mammals are also lethal because of absent expression of maternally expressed genes and excessive expression of paternally expressed genes. However, such mammals could be viable in the presence of a co-existing biparental cell lineage. Indeed, parthenogenetic and androgenetic mosaicisms have been identified in the human [2–7].

To our knowledge, parthenogenetic mosaicism has been identified in five patients [2–6]. Molecular data informative for the assessment of the generation mechanism invariably imply that parthenogenetic activation has taken place around the time of fertilization, resulting in the generation of the 46,XX parthenogenetic cell lineage by endoreplication of a female pronucleus and the biparental cell lineage by fusion of male and female pronuclei [2, 4–6]. Thus, although other mechanisms such as retention of second polar body and failure of the paternal pronucleus to duplicate, followed by endoreplication of a female pronucleus and fusion of biparentally derived pronuclei, have been assumed [6], there is no report describing such generation mechanisms. Furthermore, while such patients frequently exhibit growth failure with some Silver-Russell syndrome (SRS) features [2–6], no specific phenotype ascribed to unmasking of a recessive pathogenic variant(s) of maternal origin in the parthenogenetic cell lineage has been described, probably due to the buffering effect of a co-existing paternally derived wildtype (WT) allele or the absence of such a variant(s) in the previously reported patients.

Here, we report parthenogenetic mosaicism mediated by second polar body retention and an unmasked *PER2* (period circadian regulator 2) variant in a female with SRS and idiopathic hypersomnia (IH).

## Results

### Case report

This Japanese female was conceived naturally and was born at 42 weeks of gestation after an uncomplicated pregnancy and delivery. The parents were both 29 years of age at her birth and were non-consanguineous and healthy, with the paternal height of 168 cm (− 0.5 SD) and the maternal height of 156 cm (− 0.4 SD). The elder brother was also healthy.

**Fig. 1:**
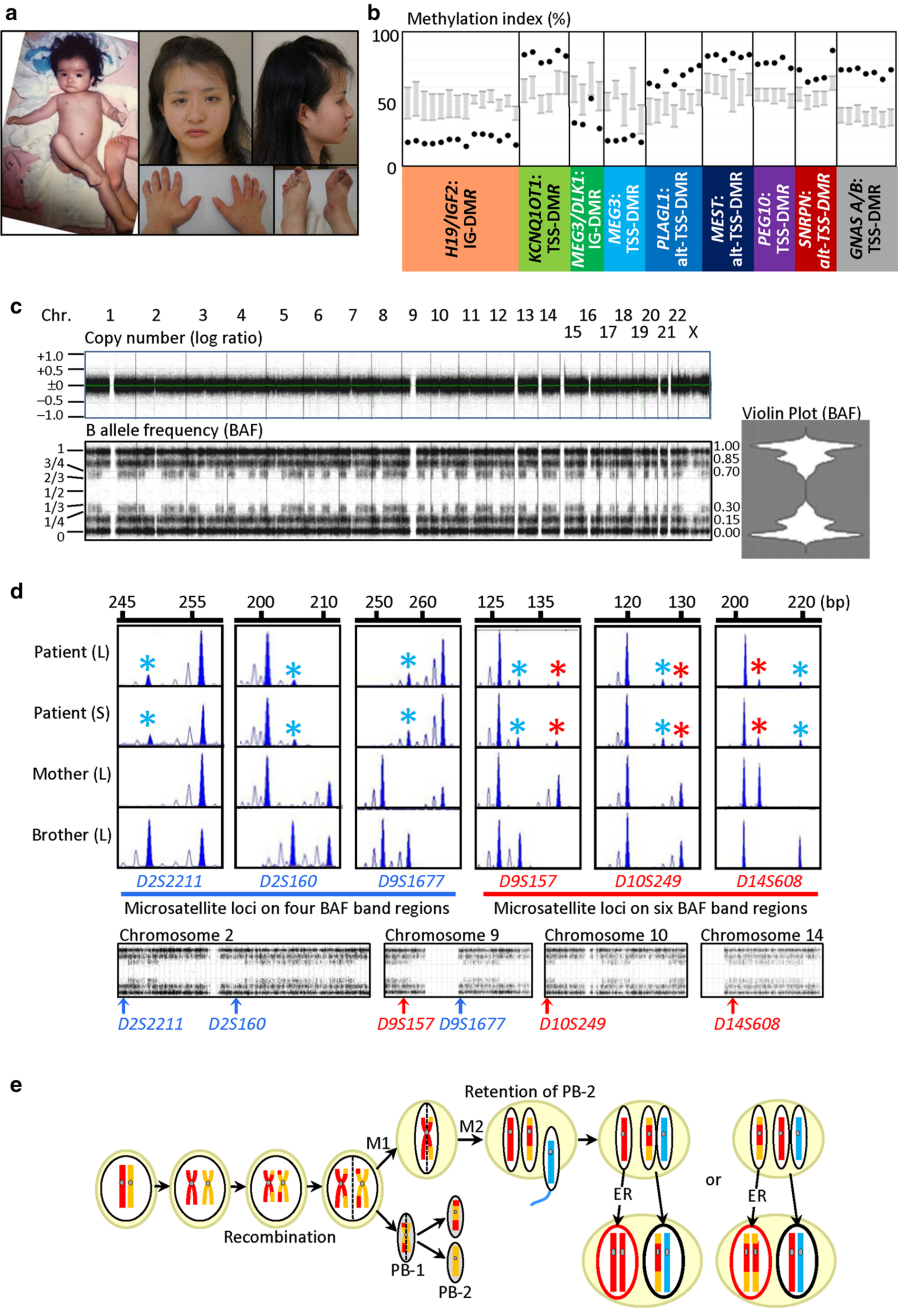
Clinical and molecular findings of this patient. **a** Photographs of this patient at one and 28 years of age. **b** Methylation indices for imprinting disease-related DMRs, obtained by pyrosequencing. Normally, the *H19*/*IGF2*:IG-DMR, *MEG3*/*DLK1*:IG-DMR, and *MEG3*:TSS-DMR are methylated after paternal transmission, and the remaining DMRs are methylated after maternal transmission. The physical positions of examined DMRs are described in Table S3. Gray vertical bars indicate the reference ranges (minimum – maximum) in 50 control subjects. **c** Array-based genomewide copy-number and BAF analyses using CytoScan HD, and violin plot for the BAFs. **d** Microsatellite analysis using leukocyte (L) and saliva cells (S). In addition to single major peaks consistent with maternal isodisomy, minor peaks of non-maternal (paternal) origin are detected for three loci on four BAF band regions (written in blue), and those of maternal and non-maternal (paternal) origin are identified for the remaining three loci on six BAF band regions (written in red). Minor peaks of maternal and non-maternal (paternal) origin are indicated with red and blue asterisks, respectively. **e** Schematic representation of the generation of the parthenogenetic 46,XX cell lineage and the biparental 46,XX cell lineage. For simplicity, the behavior of single homologous chromosomes is shown, and all homologous chromosomes act similarly. The red and orange bars indicate homologous chromosomes in the oocyte, and the blue bar denotes a homologous chromosome in the sperm. M1, meiosis 1; M2, meiosis 2; PB-1, first polar body; PB-2, second polar body; and ER, endoreplication

She exhibited SRS-compatible clinical features satisfying the Netchine-Harbison criteria [8] in infancy to early childhood (Table 1 and Fig. 1a). She also exhibited overt hypotonia and borderline developmental delay: she controlled her head at six months of age, sat without support at nine months of age, walked without support at 23 months of age, and spoke single words at two years of age. She received growth hormone therapy for short stature from 6 to 12 years of age, and gonadotropin releasing hormone analog therapy for central precocious puberty with menarche at 7.5 years of age (the menarchial age in Japanese girls, 12.25 ± 1.25 years) from 7 to 12 years of age. She also had hypersomnia from childhood and truncal obesity and oligomenorrhea from late teens.

**Table 1:** Clinical findings in six cases with parthenogenetic mosaicism

	Case 1	Case 2	Case 3	Case 4	Case 5	Case 6
<*Parthenogenetic mosaicism*>						
Karyotype	46,XX/46,XY	46,XX/46,XY/69,XXY	46,XX/46,XY	45,X/46,XX	46,XX/47,XXX	46,XX
Parthenogenetic cells	46,XX	46,XX	46,XX	46,XX	46,XX	46,XX
Non-parthenogenetic cells	46,XY	46,XY & 69,XXY	46,XY	45,X	47,XXX	46,XX
Frequency of parthenogenetic cells	> 98% (L)	~ 100% (L)	0% (L)	84% (L)	42% (Amniocytes)	70% (L)
	< 5% (SF)	~ 0% (SF)	45% (SF)	56% (Saliva)		67% (Saliva)
Generation mechanism	PA and ER	Unknown	PA and ER	PA and ER	PA and ER	PB-2 retention and ER
<*Clinical features*>						
Present age	1.2 years	Unknown	Unknown	34 years	18 weeks of gestation	28 years
Phenotypic/social sex	Male	Male	Male	Female	Unknown	Female
Prenatal growth failure	–	+	Unknown	+	+	+
Birth length — cm	Unknown	Unknown	Unknown	44.0 (− 3.1 SD)	… (Fetus)	42.0 (− 4.6 SD)
Birth weight — kg	3.36 (− 0.5 SD)	< 3rd percentile	Unknown	2.10 (− 2.9 SD)	… (Fetus)	2.41 (− 3.0 SD)
Birth OFC — cm	Unknown	50th percentile	Unknown	30.5 (− 2.3 SD)	… (Fetus)	Unknown
Postnatal growth failure	Unknown	–	Unknown	+	… (Fetus)	+
Present height — cm	Unknown	25 percentile	Unknown	125.0 (− 6.2 SD)	… (Fetus)	146.9 (− 2.1 SD)
Present weight — kg	Unknown	Unknown	Unknown	37.5 (− 2.0 SD)	… (Fetus)	60.4 (+ 0.9 SD)
Present OFC — cm	Unknown	Unknown	Unknown	51.2 (− 2.8 SD)	… (Fetus)	55.6 (− 0.1 SD)
Intellectual disability	+ (Mild)	+ (Moderate)	+	+ (DQ 56)	… (Fetus)	±
Hypotonia	Unknown	Unknown	Unknown	+	… (Fetus)	+
Disorder of sex development	+ (46,XX DSD)	+ (Genital anomalies)	Unknown	–	… (Fetus)	–
<*SRS: Netchine-Harbison scoring system features*>[7]						
Birth length and/or weight ≤ − 2 SDS	–	+ (probably)	Unknown	+	… (Fetus)	+
Relative macrocephaly at birth^a^	Unknown	+ (probably)	Unknown	+	… (Fetus)	Unknown
Postnatal height (at ~ 2 years) ≤ − 2 SDS	Unknown	–	Unknown	+	… (Fetus)	+
Prominent forehead (1–3 years)	Unknown	+ (probably)	Unknown	+	… (Fetus)	+
Body asymmetry	+	+	+	+	… (Fetus)	+
Feeding difficulties and/or low BMI	Unknown	Unknown	Unknown	Unknown	… (Fetus)	+
Other features	Footnote-1	Footnote-2	Footnote-3	Footnote-4	Footnote-5	Footnote-6
References	[2]	[3] (Patient 11)	[4] (Patient 30)	[5]	[6]	This patient

Footnote-1: Bifid uvula and submucous cleft palate. Footnote-2: Clinodactyly, syndactyly of toes, and down-turned mouth. Footnote-3: Skin pigmentation, hearing loss, and childhood obesity. Footnote-4: Triangular face, 5th finger clinodactyly, and horseshoe kidney. Footnote-5: Short nasal bone, single umbilical artery, and diaphragmatic hernia (left). Footnote-6: Bilateral myopia, left hearing loss, hypoplastic and low-set ears, micrognathia, irregular teeth, small hands and feet, 5th finger shortening and clinodactyly, big and laterally deviated toes, café au lait spots, truncal obesity, precocious puberty, and hypersomnia

OFC, occipitofrontal circumference; SDS, standard deviation score; BMI, body mass index; L, leukocytes; SF, skin fibroblasts; PA, parthenogenetic activation; ER, endoreplication; PB-2, second polar body; and DQ, developmental quotient

^a^Birth OFC SDS ≥ 1.5 above birth length or weight SDS

At 28 years of age, she was referred to us for the genetic studies of SRS. At that time, she manifested short stature, relative macrocephaly, hemihypoplasia, truncal obesity, borderline intellectual disability, and mild hypotonia, together with several other features (Table 1 and its Footnote-6 and Fig. 1a). Biochemical and radiological findings were grossly normal, except for mild fatty liver, low blood estradiol (24 pg/mL) (reference values: follicular phase 29 – 197 pg/mL, luteal phase 44 – 492 pg/mL), and hypoplastic ovaries and uterus. Her karyotype was 46,XX in all the 100 lymphocytes examined. Estrogen and progesterone replacement therapy was started, successfully inducing menses.

We performed detailed examinations for hypersomnia which was specific to her and compromised her quality of life. She had prolonged nocturnal sleep, daytime sleepiness, unrefreshing naps, and difficulty in awaking, and had no episode suggestive of cataplexy or severe snoring. Orexin concentration was 242 pg/mL (reference value, > 110 pg/mL) in the cerebrospinal fluid, and HLA typing indicated DQB1*03:01/DQB1*06:01 genotype and excluded the narcolepsy-associated DQB1*06:02 allele [9]. Polysomnography (PSG) revealed a total sleep time of 505 min (age-matched average sleep time, 460 min) with normal sleep structure, apnea-hypoxia index of 2.9/h (reference value, < 5/h), and 3% oxygen desaturation index of 5.3/hour (reference value, < 15/h). Multiple sleep latency test (MSLT) showed remarkably short sleep latency of 1.5 min, and no sleep onset rapid eye movement period (SOREMP). These findings, especially the sleep-related symptoms and the combination of sleep latency of < 8 min and no SOREMP on PSG/MSLT, indicated IH rather than sleep apnea syndrome, narcolepsy (both type 1 with cataplexy and type 2 without cataplexy), and circadian rhythm sleep-wake disorders such as advanced or delayed sleep-wake phase disorder [9–11]. After thorough consultation, she received modafinil treatment [11], which appeared effective for the first ~ two months but became obviously ineffective thereafter. Thus, it was discontinued after six months of therapy.

### Sample preparations

We obtained leukocyte gDNA samples of the patient, the mother, and the brother, and salivary cell gDNA sample of the patient. Unfortunately, paternal sample was not available, because he had been deceased.

### Methylation analysis

Pyrosequencing analysis showed abnormal, but not extremely skewed (e.g., < 10% or > 90%), methylation indices (MIs, the ratios of methylated clones) not only for SRS-related differentially methylated regions (DMRs) but also for other imprinting disease-related DMRs (Fig. 1b). The MIs were consistent with the predominance of maternally derived DMRs.

### Genomewide array-based analysis

CytoScan HD revealed no known pathogenic copy-number variant (CNV) or CNV of unknown significance absent from public databases. B allele frequency (BAF) analysis showed two types of regions for all chromosomes, i.e., one type of regions with four bands for different BAFs (0.00, ~ 0.15, ~ 0.85, and 1.00) and the other type of regions with six bands for different BAFs (0.00, ~ 0.15, ~ 0.30, ~ 0.70, ~ 0.85, and 1.00) (Fig. 1c and Additional file 1: Fig. S1). The pericentromeric portions were invariably composed of four BAF band regions.

### Microsatellite analysis

Microsatellite analysis delineated a different pattern between four and six BAF band regions (Fig. 1d and Additional file 1: Table S1). For loci on four BAF band regions (shown in blue), informative results were obtained for several loci, showing two peaks in the patient, one major peaks consistent with maternal isodisomy and one minor peaks of non-maternal (paternal) origin. For loci on six BAF band regions (shown in red), informative results were also obtained for several loci, showing three peaks in the patient, one major peaks consistent with maternal isodisomy and two minor peaks of maternal and non-maternal (paternal) origin. The frequency of the parthenogenetic cells was calculated as 70% in leukocytes and 67% in salivary cells, using informative microsatellite data for loci on four BAF band regions (e.g., *D2S2211*) (Additional file 1: Note).

### Whole exome sequencing

**Fig. 2:**
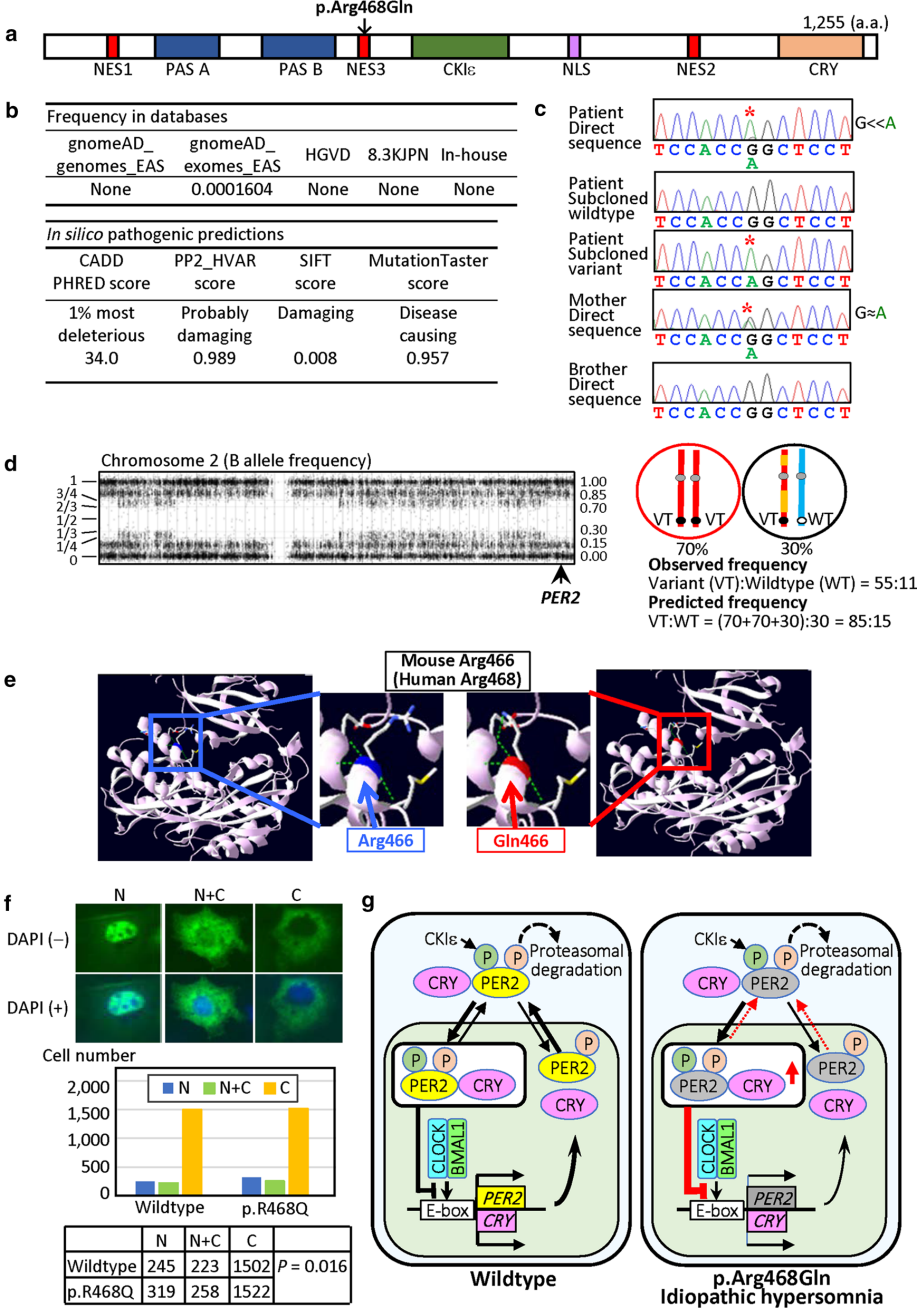
The *PER2* variant identified in this patient. **a** Structure of the PER2 protein and the position of the p.Arg468Gln. NES, nuclear export signal; PAS, PER-ARNT-SIM; CKIε, casein kinase Iε; NLS, nuclear localization signal; and CRY, cryptochrome. **b** Extreme rarity of the c.1403G>A variant in representative public databases and in-house database, and high pathogenicity of this variant predicted by different methods (for URLs, see Methods). **c** Electrochromatograms showing the maternally inherited c.1403G>A substitution (marked with red asterisks). Direct sequencing denotes that the area under curve is much larger for the variant (VT) ″A″ allele than for the wildtype (WT) ″G″ allele in the patient, while it is similar between the ″A″ and ″G″ alleles in the mother. **d** The position of *PER2* on chromosome 2 and the frequency of the variant allele. *PER2* is present on the four BAF band region, and the observed frequencies of the VT and WT alleles in *PER2* are similar to the predicted frequencies of the VT and WT alleles in a gene on the four BAF band regions (see Additional file 1: Fig. S2). **e** Protein modelling analysis. Dashed green lines indicate hydrogen bonds. **f** Subcellular distribution analysis. Shown are representative subcellular distribution patterns of the GFP-labelled p.Arg468Gln-PER2 protein, and the number of cells assessed as "N", "N + C", and "C" distribution patterns. DAPI, 4′,6-diamidino-2-phenylindole; N, nucleus-dominant distribution; N + C, both nuclear and cytoplasmic distribution; and C, cytoplasm-dominant distribution. **g** Simplified schematic representation of the circadian cycle [19, 20, 24, 25]. PER2 protein contains at least two functionally different phosphorylation sites: one hitherto unidentified site that primarily mediates proteasomal degradation (target site-1, dark orange) and the other site for the nuclear retention that is phosphorylated by CKIε (target site-2, dark green). Left part indicates the circadian cycle with WT-PER2 (highlighted with yellow). At the beginning of the circadian cycle (morning – midday), the CLOCK-BMAL1 heterodimer activates *PER2* and *CRY* expressions by binding to their regulatory elements (E-box), and PER2 with phosphorylation at the target site-1 and CRY are exported to the cytoplasm. At the later phase of the circadian cycle (afternoon – early night), PER2 with phosphorylation at the both target sites-1 and -2 forms a heterodimer with CRY, and the PER2-CRY heterodimer is imported to the nucleus where it represses the transactivation function of the CLOCK-BMAL1 heterodimer for *PER2* and *CRY*. At the end of the circadian cycle (late night), the repression function of the PER2-CRY heterodimer is decreased because of the degradation of PER2 with phosphorylation at the target site-1 in the cytoplasm. This permits the operation of the circadian rhythm. Right part indicates the circadian cycle with the p.Arg468Gln-PER2 variant (highlighted with dark gray). In this case, it is predicted that nuclear export of PER2 and PER2-CRY heterodimer is compromised (indicated with thin red dotted arrows) because of the position of the variant at NES3, leading to a relative accumulation of the PER2-CRY heterodimer in the nucleus (shown with a red vertical arrow) and, consequently, a prolonged repression by the PER2-CRY heterodimer for the CLOCK-BMAL1 transactivation function (indicated with thick red bars). This would decelerate the production of PER2 and CRY, leading to IH

Whole exome sequencing was performed, to identify an underlying genetic factor(s) for IH specific to this patient. Consequently, a missense variant on the third nuclear export signal (NES3) of *PER2* (NM_022817.2:c.1403G>A:p.(Arg468Gln)) (https://www.ncbi.nlm.nih.gov/genbank) was identified as the most likely underlying factor for IH, because of its extreme rarity in the public and in-house databases (registered in gnomeAD_exome_EAS in a heterozygous condition with the allele frequency of 0.0001604), high predicted pathogenicity, and relevance to the circadian rhythm [12] (Fig. 2a–c). This variant was detected in 55 of 66 reads (83.3%) in this patient and in 25 of 52 reads (48.0%) in the mother. The frequency was well consistent with the genomic position of *PER2* on the four BAF band region where BAF is assumed to be ~ 85% (Fig. 2d). No other rare and pathogenic variants were identified in sleep-related genes [12].

### Protein modelling analysis

In the mouse crystal structure of a PAS domain fragment (Protein Data bank ID code: 3GDI), p.Arg466Gln, which corresponds to p.Arg468Gln in the human, was evaluated to mildly affect the local protein structure and hydrogen bonds (Fig. 2e).

### Subcellular distribution analysis

Since this variant resided at NES3, we compared subcellular distribution pattern between the GFP-labelled WT-PER2 and p.Arg468Gln-PER2 proteins. The p.Arg468Gln-PER2 protein was mildly but significantly more localized to the nucleus than the WT-PER2 protein (Fig. 2f).

## Discussion

Molecular studies indicate that this patient had parthenogenetic mosaicism. It is inferred that the second polar retention occurred around the time of fertilization, resulting in the generation of the parthenogenetic cell lineage by endoreplication of a female pronucleus and the normal cell lineage by fusion of male and female pronuclei (Fig. 1e) [6] (thus, in a strict sense, this condition is neither mosaicism caused by a mitotic error of a single zygote nor chimerism resulting from the fusion of two different zygotes). In this case, this patient is assumed to have three haploid sets of maternal origin (two in the parthenogenetic cell lineage and one in the biparental cell lineage) consisting of homozygous and heterozygous regions, with pericentromeric portions being composed of homozygous regions, together with one haploid set of paternal origin (Fig. 1e). This explains why the MIs were outside the normal range but were not extremely skewed, and why this patient had not only four BAF band regions with single major microsatellite peaks of maternal origin and single minor microsatellite peaks of non-maternal (paternal) origin but also six BAF band regions with single major microsatellite peaks of maternal origin and two minor microsatellite peaks of maternal and non-maternal (paternal) origin (Additional file 1: Fig. S2). The usefulness of SNP array in the elucidation of generation mechanisms of such rare conditions has been reported previously [4, 6]. Indeed, CytoScan HD analysis has confirmed the underlying generation mechanisms in the previously reported parthenogenetic and androgenetic mosaic patients (Additional file 1: Figs. S3 and S4) [5, 7].

Several findings are notable with regard to the parthenogenetic mosaicism of this patient. First, the mosaic ratio estimated by microsatellite analysis was well consistent with the BAFs obtained by SNP array (Additional file 1: Fig. S2). This would argue for the accuracy of the molecular studies employed in this study. Second, parthenogenetic cells accounted for the majority of leukocytes, despite the apparently global negative selective pressure of the parthenogenesis [13]. This would primarily be due to the bias in the human study. Indeed, genetic abnormalities would not be identified if they are barely present in leukocytes. In addition, parthenogenetic cells might be well preserved in several tissues of subjects with hitherto unknown specific genetic and environmental factors. Third, the mosaic ratio was similar between leukocytes and salivary cells. This would probably be co-incidental, because it was rather different between the two tissues in the previously reported patient (case 4 in Table 1) [5]. Indeed, since adult saliva cells primarily consist of leukocytes of mesodermal origin and epithelial cells of ectodermal origin with a roughly similar frequency [14], the mosaic ratio could be variable between the leukocytes and salivary cells.

To date, parthenogenetic mosaicism has been identified in six subjects including this patient (Table 1) [2–6]. Although clinical information remains fragmentary, they primarily exhibit growth failure with some SRS features including hemihypoplasia. This would primarily be consistent with the occurrence of such phenotypes in upd(7)mat, upd(11)mat, upd(14)mat, upd(16)mat, and upd(20)mat [7, 15]. In this regard, hemihypoplasia would probably be due to laterality in the distribution of parthenogenetic cells, and disorders of sex development in cases 1 and 2 is explained by the co-existing 46,XY cell lineage. In addition, upd(15)mat might be relevant to the development of several features such as growth failure, intellectual disability, and hypotonia observed in Prader-Willi syndrome [16], although such non-specific features are also reported in SRS [8]. For our patient, it is notable that she exhibited SRS-compatible phenotype in infancy and central precocious puberty and truncal obesity in a later age. Since such phenotype is characteristic of Temple syndrome caused by upd(14)mat [17], this may suggest an epi-dominant clinical effect of the chromosome 14q32.2 imprinted region and a relatively high frequency of the parthenogenetic cells in tissues/organs related to the phenotype of this patient.

Notably, this patient had IH and a c.1403G>A:p.(Arg468Gln) variant in *PER2*. *PER2* is involved in the mammalian circadian clock system, together with cryptochrome (CRY), circadian locomotor output cycles kaput (CLOCK), and brain and muscle Arnt-like protein 1 (BMAL1) [18–20]. *PER2* and *CRY* are positively expressed by the CLOCK-BMAL1 heterodimer, and PER2 and CRY proteins are exported from the nucleus to the cytoplasm. Then, the PER2-CRY complex generated in the cytoplasm is imported into the nucleus to inhibit their own transcriptions by directly interacting with the CLOCK-BMAL1 complex. This feedback cycle underlies the circadian rhythm (Fig. 2g and its legend) [18–20]. In this context, the p.Arg468Gln-PER2 protein of this patient distributed more frequently in the nucleus than the WT-PER2 protein, consistent with the variant being present at NES3. This would have resulted in the reduced PER2 and CRY production via a prolonged repressing effect of the PER2-CRY dimer on the CLOCK-BMAL1 complex, leading to the development of IH as a circadian rhythm disorder (Fig. 2g) [21]. In this context, since the mother heterozygous for the c.1403G>A:p.(Arg468Gln) variant had no sleep-related phenotype, this variant would have a recessive effect. The presence of this extremely rare variant in a heterozygous condition in the gnomeAD_exome_EAS database would also support the recessive effect. It is likely, therefore, that the recessive effect of the c.1403G>A:p.(Arg468Gln) variant was unmasked in the parthenogenetic cells, and that parthenogenetic cells accounted for a considerable degree of cells in the sleep control region such as the suprachiasmatic nucleus [18]. Thus, the c.1403G>A:p.(Arg468Gln) variant would be regarded as a likely causative variant for the development of IH. In addition, since *PER2* exerts biological effects on multiple systems [20], the c.1403G>A:p.(Arg468Gln) variant might also be involved in the development of phenotypes other than IH, such as central obesity, of this patient.

Several matters would be worth pointing out for *PER2*. First, the subcellular distribution was not so markedly different, though significant, between the WT-PER2 and p.Arg468Gln-PER2 proteins. This would not be unexpected, because: (1) p.Arg468Gln was assessed to just mildly affect the local protein structure and hydrogen bonds (Fig. 2e); (2) p.Arg468Gln is an exchange between hydrophilic basic and polar amino acids, while hydrophobic amino acids (e.g., leucine and isoleucine) are likely to play a crucial role in the NES [22]; and (3) substitution of one leucine and one isoleucine residues at NES3 with hydrophobic alanine residues has exerted a significant but mild effect on the subcellular distribution as observed in this study (replacement of two leucine residues at NES1 and that of four leucine residues at NES2 with alanine residues have markedly and mildly affected the subcellular distribution, respectively) [23]. It appears therefore, that such a relatively mild alteration in the subcellular distribution can cause a clinically recognizable sleep phenotype. Second, a pathogenic p.Ser662Gly variant has been identified at the CKIε domain in a family with dominantly inherited advanced sleep-wake phase disorder (ASWPD) [24]. In contrast to the p.Arg468Gln, the p.Ser662Gly has been implicated to cause premature nuclear export of the p.Ser662Gly-PER2 protein [19, 20, 24, 25] (for details, see Additional file 1: Fig. S5). It is likely, therefore, that *PER2* variants lead to different sleep phenotypes (IH and ASWPD), probably depending on the subcellular distribution pattern of the variant PER2 proteins. Lastly, significant associations have been identified between multiple SNPs and various sleep-wake phenotypes (Additional file 1: Table S2). This indicates that *PER2* variants function not only as causative factors but also as susceptibility factors.

## Conclusions

We identified the first case of parthenogenetic mosaicism that was generated via second polar body retention and unmasked a recessive *PER2* variant for IH. This implies the presence of different generation mechanisms for parthenogenetic mosaicism and the unmasking of a maternal recessive variant by parthenogenesis.

## Methods

### Primers

Primers used are shown in Additional file 1: Table S3.

### Methylation analysis

Pyrosequencing was performed for 59 CpG sites at nine DMRs involved in the development of SRS and other human imprinting disorders, using bisulfite-treated leukocyte gDNA samples. The procedure was as described in the manufacturer′s instructions (Qiagen). Subsequently, MIs were obtained using PyroMark Q24 (Qiagen). The data from 50 healthy subjects were utilized as references.

### Genomewide array-based analysis

CNV and BAF analyses were performed using CytoScan HD (ThermoFisher Scientific) harboring more than 2.4 million CNV markers and approximately 750,000 SNP markers. The log ratio and B-allele frequency were calculated using Rawcopy R package [26]. We selected CNVs larger than 100-kb, using log-ratio thresholds of > +0.2 for gains and < −0.3 for deletions, as suggested previously [26], and these CNVs were annotated using AnnotSV (Annotation and Ranking of Human Structural Variations) (https://www.lbgi.fr/AnnotSV/runjob). We searched for known pathogenic CNVs and CNVs of unknown significance absent from multiple public databases adopted in AnnotSV. CNVs located on segmental duplications were regarded as begin CNVs. Violin plot for BAFs was made with snp.profile.Rdata by ggplot2 (https://ggplot2.tidyverse.org/reference/index.html).

### Microsatellite analysis

PCR was performed with a fluorescently labeled forward primer and an unlabeled reverse primer flanking each microsatellite locus. Subsequently, PCR products were determined for physical sizes and area under curves on an ABI PRISM 3100xl Genetic Analyzer using Gene Mapper software version 4.1 (ThermoFisher Scientific).

### Whole exome sequencing

Whole exome sequencing was carried out using SureSelect Human All Exon V6 (Agilent Technologies). Captured libraries were sequenced by NextSeq 500 (Illumina) with 150 bp paired-end reads. Exome data processing, variant calling, and variant annotation were carried out, as described previously [27]. Human GRCh37 was utilized as the reference genome, and the *PER2* variant was described according to the HGVS recommendations [28], using NM_022817.2 as the reference sequence (https://www.ncbi.nlm.nih.gov/genbank). We extracted rare variants with minor allele frequencies of < 0.005 in all the following public databases and in-house database (*n* = 218): (1) whole genome and exome data for East Asian Population in Genome Aggregation Database (gnomAD_genome_EAS and gnomAD_exome_EAS) (http://gnomad.broadinstitute.org/); (2) Human Genetic Variation Database (HGVD) (http://www.hgvd.genome.med.kyoto-u.ac.jp/); and (3) whole-genome sequences of 8380 healthy Japanese individuals and construction of the highly accurate Japanese population reference panel (8.3KJPN) (https://ijgvd.megabank.tohoku.ac.jp/). Final variants were annotated with Annovar [29].

In silico pathogenicity predictions were carried out for identified rare variants, using the following methods: (1) CADD (Combined Annotation–Dependent Depletion) (http://cadd.gs.washington.edu/); (2) PP2_HVAR (Polyphen-2 Hum Var) (http://genetics.bwh.harvard.edu/pph2/); (3) SIFT (Sorting Intolerant From Tolerant) (http://sift.jcvi.org/); and (4) MutationTaster (http://www.mutationtaster.org/). We further examined the involvement of genes with rare variants in the circadian rhythm [12].

### Sanger sequencing

Sanger direct sequencing was performed for PCR products containing identified variants on the ABI 3130xl Genetic Analyzer (ThermoFisher Scientific). To confirm the variant, the PCR products were subcloned with the TOPO TA Cloning Kit (ThermoFisher Scientific), and wildtype and variant alleles were sequenced separately.

### Protein modelling analysis

The impact of p.Arg468Gln on protein structure was examined for mouse crystal structure of a PAS domain fragment (Protein Data bank ID code: 3GDI) using Swiss PDB Viewer. The 468th Arg codon in the human corresponds to the 466th Arg codon in the mouse.

### Subcellular distribution analysis

Full-length wildtype *PER2* cDNA (WT-*PER2*) embedded in pF1K0F vector was obtained from Kazusa DNA Research Institute (Product ID FXC06329), and variant *PER2* cDNA with c.1403G>A (c.1403G>A-*PER2*) was created by site-directed mutagenesis using PrimeSTAR Mutagenesis Basal Kit (Takara Bio). cDNAs for the WT-*PER2* and c.1403G>A-*PER2* were fused to the C-terminal side of the *GFP* gene in pEF1α-EGFP vector. Transfection was performed for COS7 cells, which were seeded into 6-well dishes containing 3 mL of DMEM with 10% fetal bovine serum (3.0 × 10^5^ cells/well) and were maintained for 24 h, using lipofectamine 3000 (ThermoFisher Scientific). Transfected COS7 cells were further cultured for 24 h, and fluorescent signals were obtained using ZOE Fluorescent Cell Imager (Bio-Rad) with and without the 4,6 diamidino-2-phenylindole (DAPI) staining. Subcellular distribution patterns were evaluated as a nuclear localization pattern (N), a nuclear-cytoplasmic distribution pattern (N + C), and a cytoplasmic localization pattern (C) by a technician who did not know the purpose of this experiment. The experiments were repeated six times, and a total of ~ 2000 cells were observed for the subcellular distribution of the WT-PER2 and p.Arg468Gln-PER2 proteins. Statistical analysis of the subcellular distribution pattern between the WT-PER2 and p.Arg468Gln-PER2 proteins (2 × 3 contingency table) was performed with the two-tailed Fisher's exact probability test.

## Supplementary Information

**Additional file 1. Table S1**: The results of microsatellite analysis. **Table S2**: Association of sleep-wake phenotypes with SNPs in PER2. Table S3: Primers utilized in this study. **Fig. S1**: Array-based copy-number and BAF analyses for each chromosome, using CytoScan HD. **Fig. S2**: Parthenogenetic mosaicism mediated by the second polar body retention. **Fig. S3**: Generation mechanism of the parthenogenetic mosaicism in the patient previously described by Yamazawa et al. **Fig. S4**: Generation mechanism of the androgenetic mosaicism in the patient described by Yamazawa et al. **Fig. S5**: The p.Ser662Gly PER2 variant identified in a dominantly inherited advanced sleep-wake phase disorder (ASWPD). **Note**: Calculation of the parthenogenetic cell frequency in leukocytes and salivary cells.

## Data Availability

All data generated or analyzed during this study are available from the corresponding author on reasonable request.

## Abbreviations

BAF: B allele frequency
CNV: Copy-number variant
DMR: Differentially methylated region
IH: Idiopathic hypersomnia
MIs: Methylation indices
MSLT: Multiple sleep latency test
NES: Nuclear export signal
PER2: Period circadian regulator 2
PSG: Polysomnography
SOREMP: Sleep onset rapid eye movement period
SRS: Silver-Russell syndrome
VT: Variant
WT: Wildtype
