# Supplementary material for: Parthenogenetic mosaicism: generation via second polar body retention and unmasking of a likely causative PER2 variant for hypersomnia
Source: Clin Epigenetics. 2021 Apr 7;13:73. doi: 10.1186/s13148-021-01062-0 (PMC8028705; doi:10.1186/s13148-021-01062-0)
Supplement: Supplementary file 1 — Additional file 1. Table S1: The results of microsatellite analysis. Table S2: Association of sleep-wake phenotypes with SNPs in PER2. Table S3: Primers utilized in this study. Fig. S1: Array-based copy-number and BAF analyses for each chromosome, using CytoScan HD. Fig. S2: Parthenogenetic mosaicism mediated by the second polar body retention. Fig. S3: Generation mechanism of the parthenogenetic mosaicism in the patient previously described by Yamazawa et al. Fig. S4: Generation mechanism of the androgenetic mosaicism in the patient described by Yamazawa et al. Fig. S5: The p.Ser662Gly PER2 variant identified in a dominantly inherited advanced sleep-wake phase disorder (ASWPD). Note: Calculation of the parthenogenetic cell frequency in leukocytes and salivary cells. [file 13148_2021_1062_MOESM1_ESM.pdf]

Supplementary Materials

Additional file 1: Table S1. The results of microsatellite analysis

Additional file 2: Table S2. Association of sleep-wake phenotypes with SNPs in *PER2*

Additional file 3: Table S3. Primers utilized in this study

Additional file 4: Figure S1. Array-based copy-number and BAF analyses for each chromosome, using CytoScan HD

Additional file 5: Figure S2. Parthenogenetic mosaicism mediated by the second polar body retention

Additional file 6: Figure S3. Generation mechanism of the parthenogenetic mosaicism in the patient previously described by Yamazawa et al.

Additional file 7: Figure S4. Generation mechanism of the androgenetic mosaicism in the patient described by Yamazawa et al.

Additional file 8: Figure S5. The p.Ser662Gly *PER2* variant identified in a dominantly inherited advanced sleep-wake phase disorder (ASWPD)

Additional file 9: Supplementary Note. Calculation of the parthenogenetic cell frequency in leukocytes and salivary cells

Supplementary Table S1. The results of microsatellite analysis

Locus	Patient	Mother	Brother	BAF bands*
D1S2841	276	276	276	6
D1S207	(242)/(250)/252	242/252	242/252	6
D2S2211	(248)/256	256	248/256	4
D2S160	201/(205)	201/211	205/211	4
D3S1263	(235)/(237)/245	235/245	235/237	6
D3S1292	149	149	141/149	4
D4S1572	129/(149)	129/149	129/149	4
D4S1535	184/(188)/(190)	184/188	188/190	6
D5S407	118	118	118	6
D5S400	217/(221)/(223?)	217/223	221/223	6
D6S257	174/(176)	174/176	156/174	4
D6S434	193/(197)	193	193/209	6
D7S672	130/(132)	130/138	138/140	4
D7S802	144	144	144	4
D7S1523	(359)/381	381	351/381	6
D8S277	161/(163)	161	161/163	4
D8S284	283	283/285	285	6
D9S157	126/(130)/(138)	126/138	126/130	6
D9S1677	(256)/264	250/264	250/256	4
D10S249	118/(126)/(130)	118/130	118/130	6
D10S537	146/(148)	146/148	146	6
D11S4046	(175)/195	175/195	175/195	4
D11S4088	214/(232)	214/228	228/232	4
D11S1356	192/(212)	192	192/212	6
D12S99	208/(218)	208/218	210/218	6
D12S1617	252/(254)	252/254	252/254	6
D13S153	215/(219)	215	215/219	6
D13S285	(89)/(95)/97	95/97	89/95	6
D14S72	162/(256)	256/262	256	4
D14S608	203/(207)/(219)	203/207	203/219	6
D14S980	165/(179)/(181)	165/181	167/181	6
D14S292	107/(113)	105/107	107/113	4
D15S128	(198)/200	200/204	204/210	4
D15S205	145/(155)	145	145/155	6
D16S514	(127)/129	117/129	117/121	4
D16S3091	114/(122)	114/122	112/122	4
D17S831	236	234/236	230/234	6
D17S928	148	146/148	146/148	6
D18S452	121/(123)/129	121/129	121/123	4
D18S53	169/(173)	169/177	169/173	4
D19S209	(257)/265	257/265	255/265	6
D19S226	232/(242)/(244)	232/244	242/244	4
D20S195	248/(252)	248/252	252	4
D20S171	134/(136)	134/136	132/136	6
D21S1252	(236)/244	244	236/244	6
D21S266	(151)/167/(169)	167/169	151/169	6
D22S315	191/(193)	187/191	191/193	6
D22S274	(201)/207	201/207	195/207	6
DXS991	283/(285)	283/285	285	4
DXS1001	(201)/207/(209)	201/207	201	6

The Arabic numbers represent the sizes of the PCR products in bp.
The numbers in parentheses of the patient indicate the PCR product sizes of the minor peaks.
BAF, B-allele frequency.
* The BAF band number of the regioins where the microsatellite loci are present.

Supplementary Table S2. Association of sleep-wake phenotypes with SNPs in *PER2*

Phenotype	SNP		Genomic position*		Reference
Chronotype	rs80261926	C>A,T	2:239152870	3_prime_UTR_variant	[1]
Idiopathic hypersomnia	rs76355956	C>T	2:239157708	Val205Met	[2]
Delayed sleep-wake phase disorder	rs76355956	C>T	2:239157708	Val205Met	[2]
Morningness	rs35333999	C>T	2:239161957	p.Val903Ile	[3]
Ease of getting up in the morning	rs35333999	C>T	2:239161957	p.Val903Ile	[3]
Morning vs. evening chronotype	rs35333999	C>T	2:239161957	p.Val903Ile	[4]
Chronotype	rs35333999	C>T	2:239161957	p.Val903Ile	[4]
Chronotype	rs35333999	C>T	2:239161957	p.Val903Ile	[5]
Ease of getting up in the morning	rs11629830	C>T	2:239177686	Intron_variant	[3]
Morningness	rs77942338	T>C	2:239194693	Intron_variant	[3]
Chronotype	rs58574366	G>A	2:239194837	Intron_variant	[1]
Chronotype	rs80271258	C/T	2:239311505	5_prime_UTR_variant	[5]
chronotype	rs75804782	T>C	2:239316043	5_prime_UTR_variant	[6]
Morningness	rs55694368	G>T	2:239317692	5_prime_UTR_variant	[7]

* According to GRCh37/h19.

References

1. Jones SE, Lane JM, Wood AR,van Hees VT, Tyrrell J, Beaumont RN, et al. Genome-wide association analyses of chronotype in 697,828 individuals provides insights into circadian rhythms. Nat Commun 2019;10:343.
2. Miyagawa T, Hida A, Shimada M, Uehara C, Nishino Y, Kadotani H, et al. A missense variant in PER2 is associated with delayed sleep-wake phase disorder in a Japanese population. J Hum Genet 2019;64:1219-1225.
3. Jansen PR, Watanabe K, Stringer S, Skene N, Bryois J, Hammerschlag AR, et al. Genome-wide analysis of insomnia in 1,331,010 individuals identifies new risk loci and functional pathways. Nat Genet 2019;51:394-403.
4. Lane JM, Vlasac I, Anderson SG, Kyle S, Dixon WG, Bechtold DA, et al. Genome-wide association analysis identifies novel loci for chronotype in 100,420 individuals from the UK Biobank. Nat Commun 2016;7:10889.
5. Chang AM, Duffy JF, Buxton OM, Lane JM, Aeschbach DA, Andersen C, et al. Chronotype genetic variant in PER2 is associated with intrinsic circadian period in humans. Sci Rep 2019;9:5350.
6. Jones SE, Tyrrell J, Wood AR, Beaumount RN, Ruth KS, Tuke MA, et al. Genome-wide association analyses in 128,266 individuals identifies new morningness and sleep duration loci. PLoS Genet 2016;12:e1006125.
7. Hu Y, Shmygelska A, Tran D, Eriksson N, Tung JY, Hinds DA. GWAS of 89,283 individuals identifies genetic variants associated with self-reporting of being a morning person. Nat Commun 2016;7:10448.

Supplementary Table S3. Primers utilized in this study.

**<Pyrosequencing>**	Forward (5' → 3')	Reverse (5' → 3')	Sequence primer
PLAGL1: alt-TSS-DMR	GGGGTAGTYGTGTTTATAGTTTAG	biotin-CCCAAACACCTACCCTAC	GGGTAGTYGTGTTTATAGTTTAGT
6q24.2	ch6: 144329336-144329359	ch6: 144329214-144329231	ch6: 144329335-144329358
PEG10: TSS-DMR	AGAAATTTGATTGYGTTTTGAGGAGAAT	biotin-ACAAAAAAAATAAAATCCCACACCTAAA	AGTTTGGYGAAAGGTT
7q21.3	chr7: 94285716-94285743	chr7:94285843-94285869	chr7: 94285762-94285777
MEST: alt-TSS-DMR	GTGTGGTTGGYGGTTTTGGGATTA	biotin-ACACCCCCTCCTCAAATA	TGTTTTTGGGYGAAAATTTTAT
7q32.2	ch7: 130132206-130132229	ch7: 130132332-130132348	ch7: 130132276-130132297
H19/IGF2: IG-DMR	TTTGGGAGAGTTTGTGAGG	CCCCAAACCRATTCCCATCCAATTA	GGTAATATGYGGTTTTTAGATAGG
11p15.5	chr11:2019763-2019781	chr11:2019579-2019603	chr11:2019689-2019712
	GTTYGGGGGTTTTTGTATAGTATATGGGT	biotin-TCCCATAAATATCCTATTCCCAAATAAC	GGTTGTAGTTGTGGAAT*
	ch11:2021295-2021315	ch11:2021072-2021099	ch11:2021221-2021237
	GTTYGGGGGTTTTTGTATAGTATATGGGT	biotin-TCCCATAAATATCCTATTCCCAAATAAC	GTTTTAATTGGGGTT*
	ch11:2021295-2021315	ch11:2021072-2021099	ch11:2021154-2021168
	GGGTTTTYGGAGGTTTTTTGGGAATA	ACTTAAATCCCAAACCATAACAC	GGAATAGGAYGTTTATGGGAG
	chr11:2023332-2023357	chr11:2023224-2023246	crh11:2023308-2023328
KCNQ1OT1: TSS-DMR	GGATTTAGAATTAYGATGYGGATTTTA	biotin-TCCCATCTACACCTTATAAACA	TTTTGAATTATTATGAGAATTATAG
11p15.5	ch11: 2720333-2720359	ch11: 2720466-2720487	ch11: 2720383-2720407
MEG3/DLK1:I G-DMR	ATTTGGTATTTGTAGTTTATGTTAAGATG	biotin-AATCAAAACAACTCAAATCCTTTATAAC	AATTGGGTTTGTTAGTAG
14q32.2	ch14: 101275613-101275642	ch14: 101275749-101275776	ch14: 101275685-101275702
MEG3: TSS-DMR	TTGTGTTTGAATTTATTTTGTTT	biotin-CCCCAAATTCTATAACAAATTACTCT	GTGTTTGAATTTATTTTGTTT
14q32.2	ch14: 101292170-101292192	ch14: 101292311-101292336	ch14: 101292172-101292192
SNURF: TSS-DMR	GTTATGGTAGTGGATTAGGGGGATGA	biotin-CCTTCCCCCTACCTCCCA	ATAGTGGTGGGGGTT
15q11.2	ch15:25200788-25200813	ch15:25200880-25200897	ch15:25200823-25200837
GNAS A/B: TSS-DMR	GGGATATTTGAGATTTTGAAAGAA	biotin-AATACAAAACCTCCCCTACT	GTTATTTTTTTTATTTGGGAGGA
20q13.32	chr20:57463531-57463555	chr20:57463727-57463746	chr20:57463630-57463652
**<Microsatellite analysis>**	Forward (5' → 3')	Reverse (5' → 3')	Genomic location
D1S2841 (1p31.1)	CCTTCTAGTGAAAATCCTCTG	TGGCTATAAATACCTTGATGC	chr1:79387295-79587659
D1S207 (1p31.1)	CACTTCTCCTTGAATCGCTT	GCAAGTCCTGTTCCAAGTCT	chr1:82543444-82543589
D2S2211 (2p25.1)	TGGGTTAGTCATCAAGGGA	GATTCAGGAGTCTGGGAAGT	chr2:7471380-7471633
D2S160 (2q13)	TGTACCTAAGCCCACCCTTTAGAGC	TGGCCTCCAGAAACCTCCAA	chr2:112998491-112998700
D3S1263 (3p25.3)	CTGTTGACCCATTGATACCC	TAAAATCACAGCAGGGGTTC	chr3:11517252-11517482
D3S1292 (3q22.1)	TGGCTTCATCACCAGACC	CAGATTCAAGAGGCACTCCA	chr3:131630365-131630522
D4S1572 (4q24)	AGACTCTAAAGATATGGTGATTTGC	TCTGATTGATTTTATGTGTGCC	chr4:103770057-103770203
D4S1535 (4q35.1)	ACTTGTGATATATACCTGCCG	TGTGAGAGCAGAATGTTGAG	chr4:185235908-185236098
D5S407 (5q11.2)	TGGTTTAGAGAATTTGCCCC	CTGTGATTTGTTGTTCATTGGAAGT	chr5:55994758-55994904
D5S400 (5q34)	GCCTGGCTGATAGAATGAGA	TTCCTAATTTGCTGGCTTCC	chr5:168442897-168443128
D6S257 (6p12.1)	GAGAACTCGTCCTTTGGTCC	TGAGAAAATGTTCAGGCTAAAGATA	chr6:55818437-56018768
D6S434 (6q16.3)	CAGGTAGTCCCCCAAAATCA	AGCTCAGGGCTTATGCCAGT	chr6:102435944-102436174
D7S672 (7q11.22)	ACATGAAGGTCTACCAGTAGCC	CACTTTGGTTGGAGCAAGG	chr7:71862617-71862660
D7S802 (7q21.11)	AAATCTATGACTCACTGG	ATATCTCACAAGGTTTCTC	chr7:81537757-81537902
D7S1523 (7q36.1)	AGAGGTTGTGGTGAACCGA	ATAGCAGCTCCCCACTTGT	chr7:151230441-151230753
D8S277 (8p23.1)	CCAGGTGAGTTTATCAATTCCTGAG	TGAGAGGTCTGAGTGACATCCG	chr8:6516725-6516870
D8S284 (8q24.22)	GGGCATGTTACTGCATGTC	TTTGAACACAGGTCTGCCA	chr8:131511698-131511966
D9S157 (9p22.2)	AGCAAGGCAAGCCACATTTC	TGGGGATGCCCAGATAACTATATC	chr9:17628302-17628512
D9S1677 (9q31.3)	CATTTGATGAATATCTGAGGGA	ACATAGCAAGGCCCCA	chr9:111937590-111937847
D10S249 (10p15.3)	AACTGGTTTTGGTAGTGAGA	GAGGTGCCCGCTAGTA	chr10:280898-281015
D10S537 (10q22.1)	CCTACTGTGCCTGGCTAGA	ATTTGGATGAAACCCACG	chr10:72395349-72395474
D11S4046 (11p15.5)	ACTCCAGCCTGGGAAAC	TGATAGACACACCCATTGC	chr11:1963642-1963832
D11S4088 (11p15.5)	GGGCAGAGGCAGTGGAG	GCATGTTTCGGGGGTG	chr11:2754951-2755157
D11S1356 (11q23.3)	GTTGCTCATCTGTTGCTCA	ACCTGCCCTGACTTGC	chr11:117915985-117916099
D12S99 (12p13.31)	GGCAGAAGTGCCTGGG	TCGAGGGTGCAGGTGG	chr12:5564771-5564899
D12S1617 (12p12.1)	AGCCTGAGGGGCCACAT	TGGGCAACTTGGATAAGAAACA	chr12:25100032-25100288
D13S153 (13q14.2)	AGCATTGTTTCATGTTGGTG	CAGCAGTGAAGGTCTAAGCC	chr13:48890820-48890954
D13S285 (13q34)	ATATATGCACATCCATCCATG	GGCCAAAGATAGATAGCAAGGTA	chr13:112795433-112795528
D14S72 (14q11.2)	TGTAAAGTTTTGTACATGGTGTAAT	TCCTAACATTCTGCTACCCA	chr14:21371011-21371277
D14S608 (14q12)	TAAAGGTTTATCCATGCTGTAGC	ACGTGGTACAGGTAGATAAATGG	chr14:28849444-28849649
D14S980 (14q22.3)	CTGGGCAACAAGAGTGG	GAAGCGGGACAATTCTCTAAG	chr14:57152567-57152726
D14S292 (14q32.33)	CTGTGTGGTGCATCAATG	CATGAAGGCAGCCTCA	chr14:104596816-104596932
D15S128 (15q11.2)	GCTGTGTGTAAGTGTGTTTTATATC	GCAAGCCAGTGGAGAG	chr15:25130800-25130998
D15S205 (15q25.2)	CTTAATGGTTTGGCAGGATA	AGCTTAAAANCAAAATCTCCC	chr15:84230721-84230880
D16S514 (16q21)	CTATCCACTCACTTTCCAGG	TCCCACTGATCATCTTCTC	chr16:62338076-62338196
D16S3091 (16q23.3)	GGGAGATAGCCTTAAACTTTCTTAC	TGTTGCTAATAACACTAGGCCA	chr16:82980565-82980683
D17S831 (17p13.3)	CGCCTTTCCTCATACTCCAG	GCCAGACGGGACTTGAATTA	chr17:1910488-1910717
D17S928 (17q25.3)	TAAAACGGCTACAACACATACA	ATTTCCCCACTGGCTG	chr17:80252880-80253028
D18S452 (18p11.31)	ATAAAAGTTGCTTCCTGGGG	TCTCCTAAATAACCGCTGGC	chr18:5829622-5829746
D18S53 (18p11.21)	GGTCACCTACAACTTTGGATG	TGCATGTAAATATCAGAGTCTGTT	chr18:11492737-11492915
D19S209 (19p13.3)	TTCATTCACAAATCNATGGC	CTGGAGAGCATAGACGNAGA	chr19:3214329-3414596
D19S226 (19p13.12)	CCAGCAGATTTTGGTGTTGTCTA	GGTCCAGGATTTGAACTAAAGCA	chr19:14633406-14633619
D20S195 (20q11.21)	GTACCTCCTCCAGGCTTC	AGGGGTGTATGTGTGCAT	chr20:31825688-31825948
D20S171 (20q13.32)	TATAGGTGAGGACCCTGAGG	ACACCAAGCCATGTAACCTG	chr20:57808030-57808168
D21S1252 (21q22.13)	TCTGTCTTTGTCTCACTATCTG	GCAATGCTCTGTGGCT	chr21:37826885-37827131
D21S266 (21q22.3)	GGGGACATTGAGTCATCACA	AGGCAAATGAAGACCTGAAC	chr21:42684564-42684717
D22S315 (22q12.1)	TGCCTATTAAACTCTCCACTCCTTA	GCATTATGATTCATTTCTCACAGA	chr22:26015881-26016065
D22S274 (22q13.31)	GTCCAGGAGGTTGATGC	AGTGCCCATTTCTCAAAATA	chr22:45269152-45269357
DXS991 (Xp11.21)	ACTTCAACCACAGAAGCCTC	ATCATTTGAGCCAATTCTCC	chrX:55519052-55519331
DXS1001 (Xq24)	TACAAGTAACCCTCGTGACA	GTTATGGAATCAATCCAAGTG	chrX:119836668-119836873
**<Sanger sequencing>**	Forward (5' → 3')	Reverse (5' → 3')	
PER2 -p.Arg468Gln	CAGGCAGCATGTAGTGGCTA	GCTACCTGGGAGGAGGACAT	
**<Mutagenesis>**	Forward (5' → 3')	Reverse (5' → 3')	
PER2 -p.Arg468Gln	ATCCACCAGCTCCTGCTGCAGCCCGTC	CAGGAGCTGGTGGATCTGCTCTGTGAG	

Y: C or T (pyrimidine); and R: A or G (purine).

Ghenomic locations are based on GRCh37/h19.

* Two sequence primers have been utilized for this region.

Chromosome 1

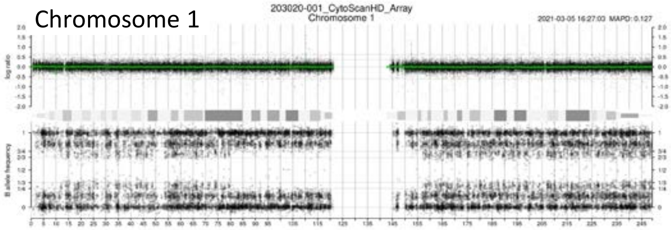

Chromosome 2

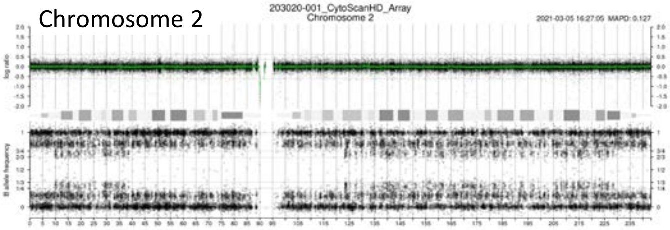

Chromosome 3

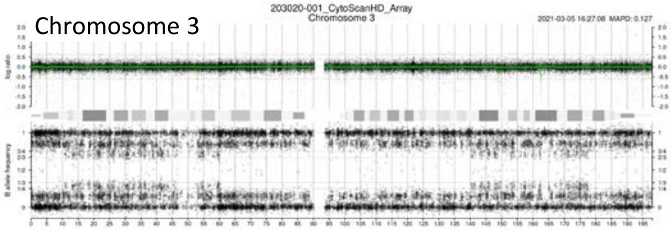

Chromosome 4

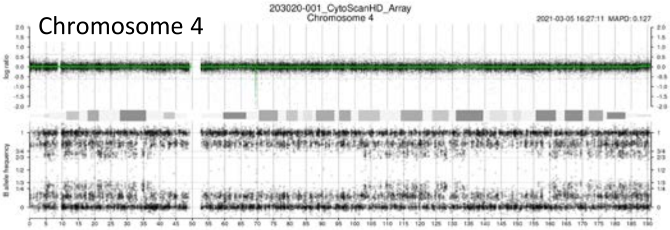

Chromosome 5

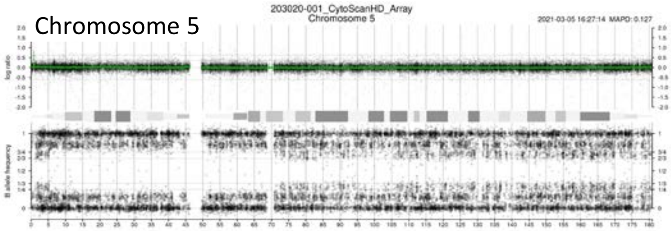

Chromosome 6

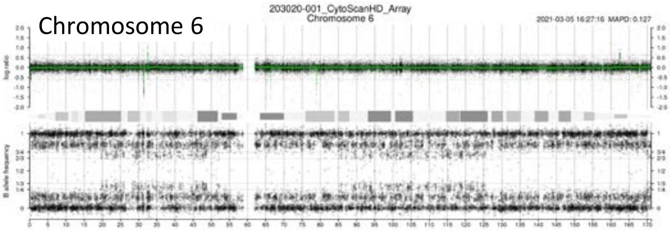

Chromosome 7

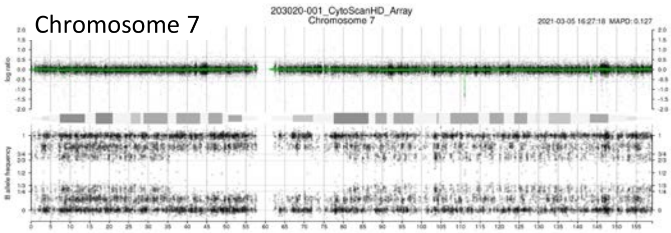

Chromosome 8

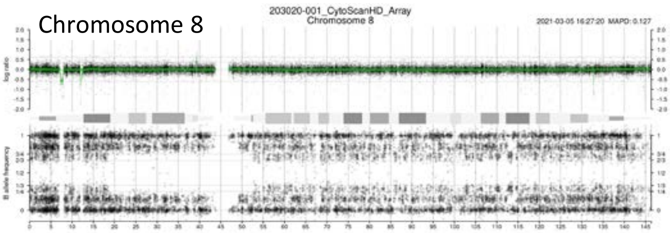

Chromosome 9

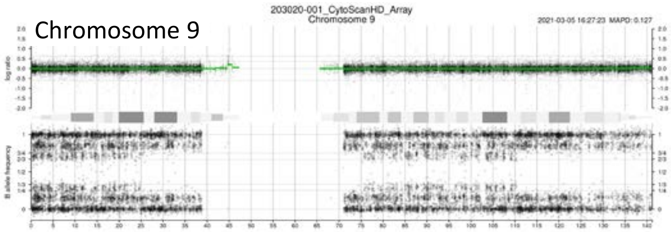

Chromosome 10

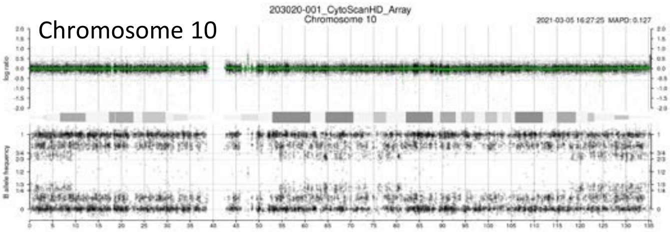

Chromosome 11

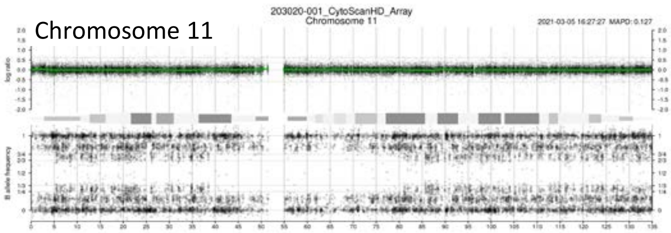

Chromosome 12

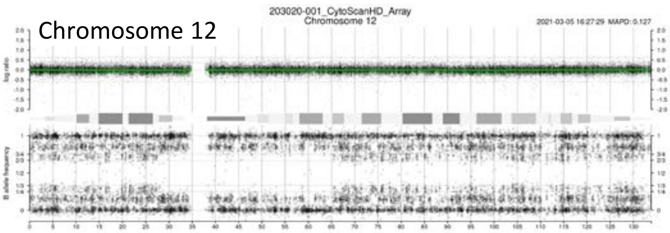

Chromosome 13

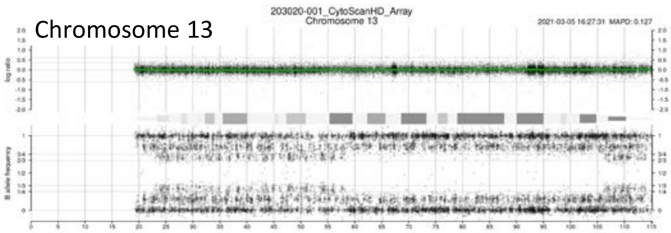

Chromosome 14

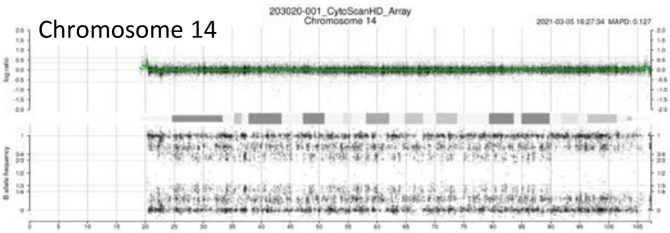

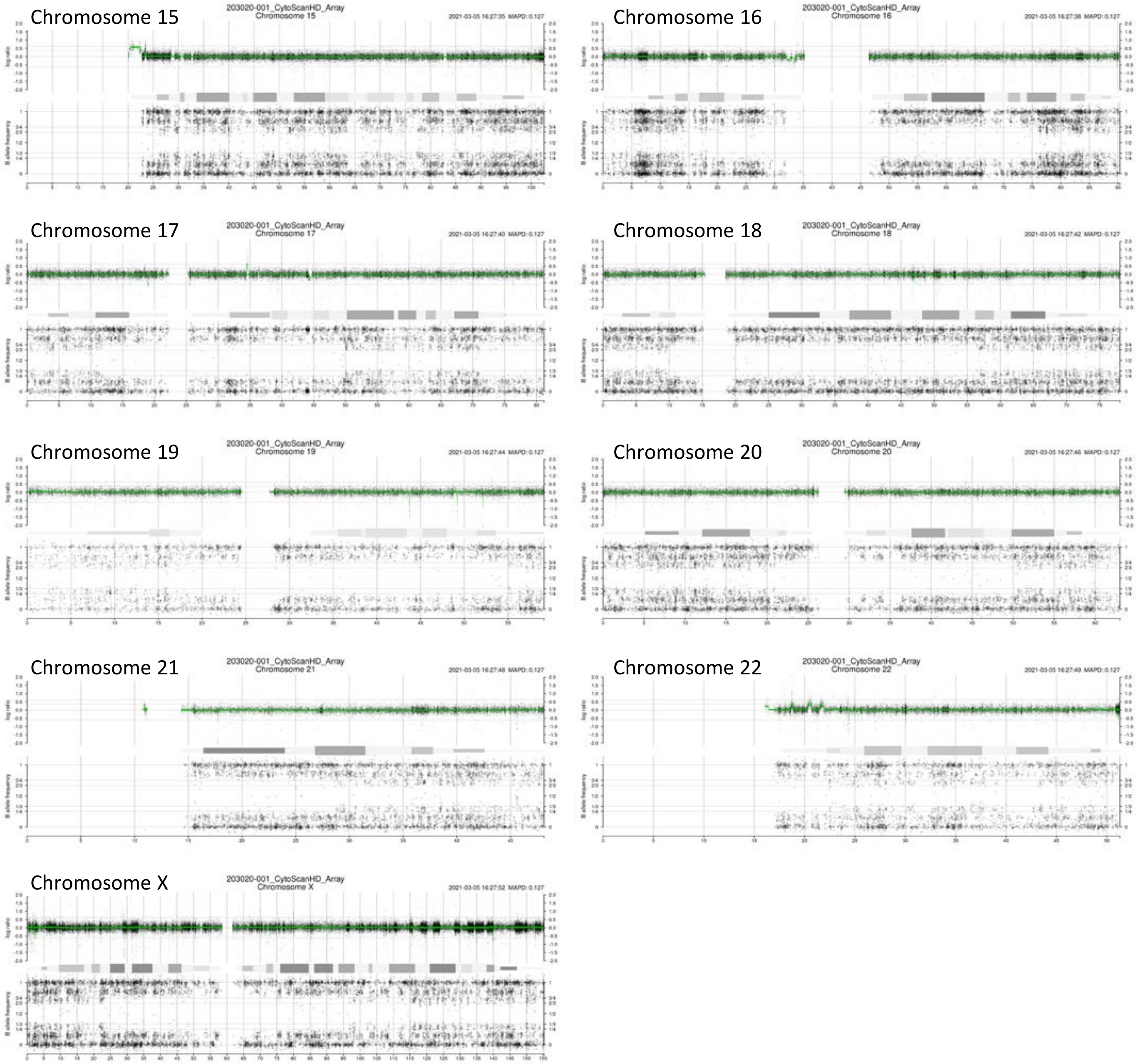

Supplementary Figure S1. Array-based copy-number and BAF analyses for each chromosome, using CytoScan HD.

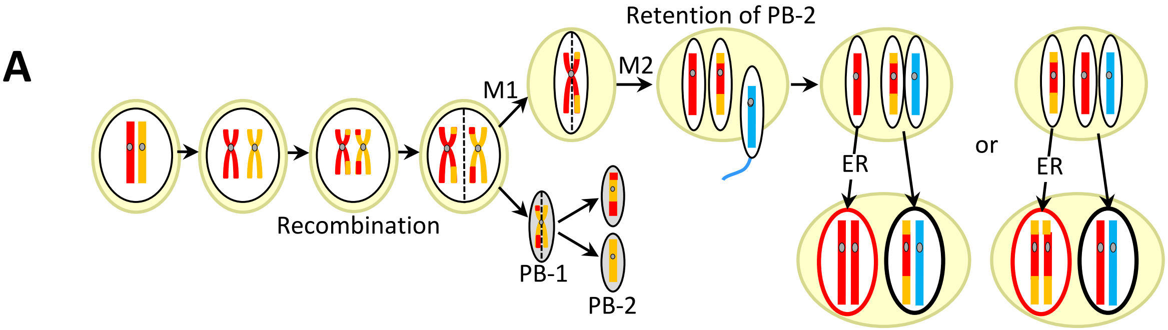

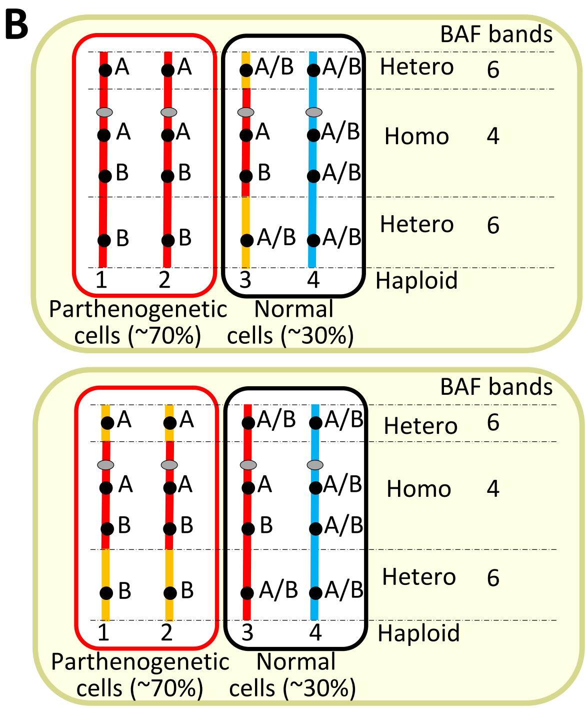

Allele numbers in 100 leukocytes				
Haploid 1	Haploid 2	Haploid 3	Haploid 4	BAF
Regions heterozygous between haploids 1 and 2 and haploid 3				
A (70)	A (70)	A (30)	A (30)	0.00 (0/200)
		A (30)	B (30)	0.15 (30/200)
		B (30)	A (30)	0.15 (30/200)
		B (30)	B (30)	0.30 (60/200)
B (70)	B (70)	A (30)	A (30)	0.70 (140/200)
		A (30)	B (30)	0.85 (170/200)
		B (30)	A (30)	0.85 (170/200)
		B (30)	B (30)	1.00 (200/200)
Regions homozygous between haploids 1 and 2 and haploid 3				
A (70)	A (70)	A (30)	A (30)	0.00 (0/200)
		A (30)	B (30)	0.15 (30/200)
B (70)	B (70)	B (30)	A (30)	0.85 (170/200)
		B (30)	B (30)	1.00 (200/200)

A: reference allele; B: non-reference allele.

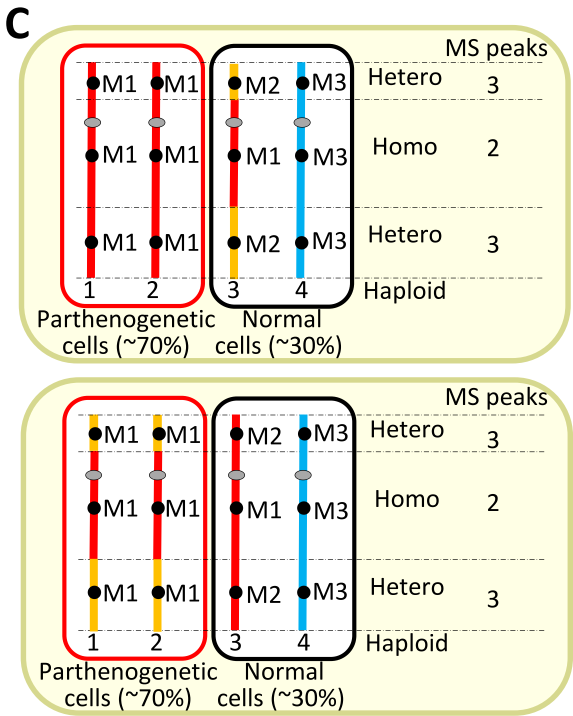

Supplementary Figure S2. Parthenogenetic mosaicism mediated by the second polar body retention.

A. Schematic representation of the generation of the parthenogenetic 46,XX cell lineage and the biparental 46,XX cell lineage. For simplicity, the behavior of single homologous chromosomes is shown, and all homologous chromosomes act similarly. The red and orange bars indicate homologous chromosomes in the oocyte, and the blue bar denotes a homologous chromosome in the sperm. M1, meiosis 1; M2, meiosis 2; PB-1, first polar body; PB-2, second polar body; and ER, endoreplication.
B. Evaluation of B-allele frequencies (BAFs). Six BAF bands can be identified for regions heterozygous between the maternally derived two haploid sets in the parthenogenetic cells and the maternally inherited single haploid set in the normal cells, whereas four BAF bands can be detected for regions homozygous between the maternally derived two haploid sets in the parthenogenetic cells and the maternally inherited single haploid set in the normal cells. The BAFs predicted from the mosaic ratio between parthenogenetic cells and normal cells (~70%:30%; see Supplementary-Note) (summarized in right Table) are well consistent with those actually obtained by CytoScan HD analysis (Figure 1C).
C. Assessment of microsatellite (MS) data. Single major peaks of maternal origin and two minor peaks of maternal and paternal origin can be delineated for regions heterozygous between the maternally derived two haploid sets in the parthenogenetic cells and the maternally inherited single haploid set in the normal cells when the MS repeat number is different between M1, M2, and M3, whereas single major peaks of maternal origin and single minor peaks of paternal origin can be detected for regions homozygous between the maternally derived two haploid sets in the parthenogenetic cells and the maternally inherited single haploid set in the normal cells when the MS repeat number is different between M1 and M3.

Parthenogenetic mosaic female (Yamazawa K, et al. Parthenogenetic chimaerism/mosaicism with a Silver-Russell Syndrome-like Phenotype. J Med Genet 2010;47:782–785).

Summary of the previous data

- Karyotype: 45,X[3]/46,XX[47].
- Microsatellite analysis: Major peaks consistent with maternal uniparental isodisomy and minor peaks of non-maternal (paternal) origin for at least one locus on each autosome, and major peaks consistent with maternal uniparental isodisomy alone for all the 30 loci on the X chromosome.
- Y-chromosome: Undetected by PCR analysis for six loci.
- Parthenogenetic cells: 84% in leukocytes (estimated by microsatellite analysis).

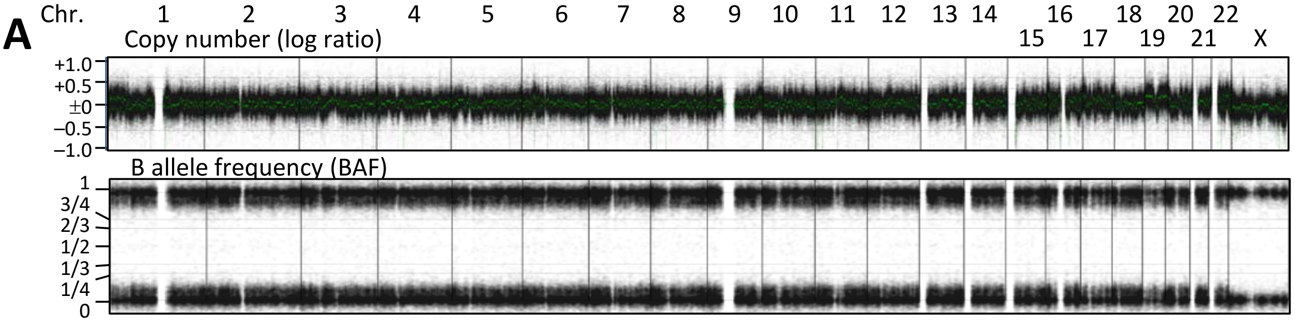

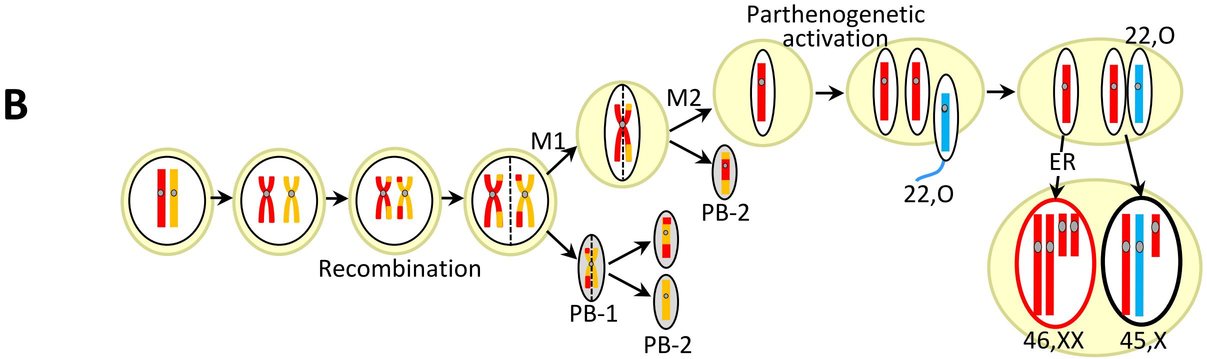

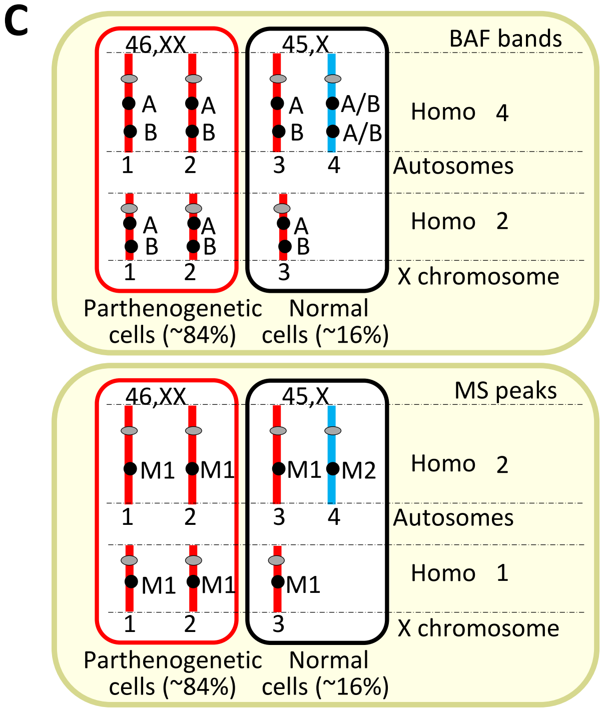

Allele numbers in 100 leukocytes				
Haploid 1	Haploid 2	Haploid 3	Haploid 4	BAF
All regions are homozygous between haploids 1–3				
<Autosomes>				
A (84)	A (84)	A (16)	A (16)	0.00 (0/200)
		A (16)	B (16)	0.08 (16/200)
B (84)	B (84)	B (16)	A (16)	0.92 (184/200)
		B (30)	B (30)	1.00 (200/200)
<X chromosome>				
A (84)	A (84)	A (16)	…	0.00 (0/184)
B (84)	B (84)	B (16)	…	1.00 (184/184)

A: reference allele; B: non-refernce allele (B-allele).

Supplementary Figure S3. Generation mechanism of the parthenogenetic mosaicism in the patient previously described by Yamazawa et al.

A. Array-based genomewide copy-number and B-allele frequency (BAF) analyses with CytoScan HD. Since leukocyte genomic DNA has been used up, genomic DNA extracted from a lymphoblastoid cell line has been utilized. Copy numbers are apparently normal for autosomes and slightly decreased for the X chromosome. Four BAF bands are identified for autosomes and two BAF bands are detected for the X chromosome.

B. Schematic representation of the generation of the parthenogenetic mosaicism. For simplicity, the behavior of single homologous autosomes and X chromosome is shown, and all homologous autosomes act similarly. It is inferred that a parthenogenetic activation took place around the time of fertilization of a sperm missing a sex chromosome (22,O), resulting in the generation of the 46,XX parthenogenetic cell lineage by endoreplication of a female pronucleus and the 45,X cell lineage with biparentally derived autosomes and a maternally derived single X chromosome by union of male and female pronuclei.

C. Evaluation of BAFs and microsatellite (MS) data. It is assumed that the maternally derived two haploid sets in the parthenogenetic cells and the maternally inherited single haploid set in the biparental cells are identical, and that the paternally inherited haploid set in the biparental cells is devoid of a sex chromosome. This would produce four BAF bands for the autosomes and two BAF bands for the X chromosome. Similarly, this would generate single major MS peaks of maternal origin and single minor MS peaks of paternal origin for autosomes when the MS repeat number is different between M1 and M2, and single MS peals only for the X chromosome.

Androgenetic mosaic female (Yamazawa K, et al. Androgenetic/biparental mosaicism in a girl with Beckwith-Wiedemann syndrome-like and upd(14)pat-like phenotypes. J Hum Genet 2011;56:91–93)

Summary of the previous data

- Karyotype: 46,XX[50].
- Microsatellite analysis: Major peaks consistent with paternal uniparental isodisomy and minor peaks of maternal origin for at least one locus on each chromosome.
- Androgenetic cells: 91% in leukocytes (estimated by microsatellite analysis).

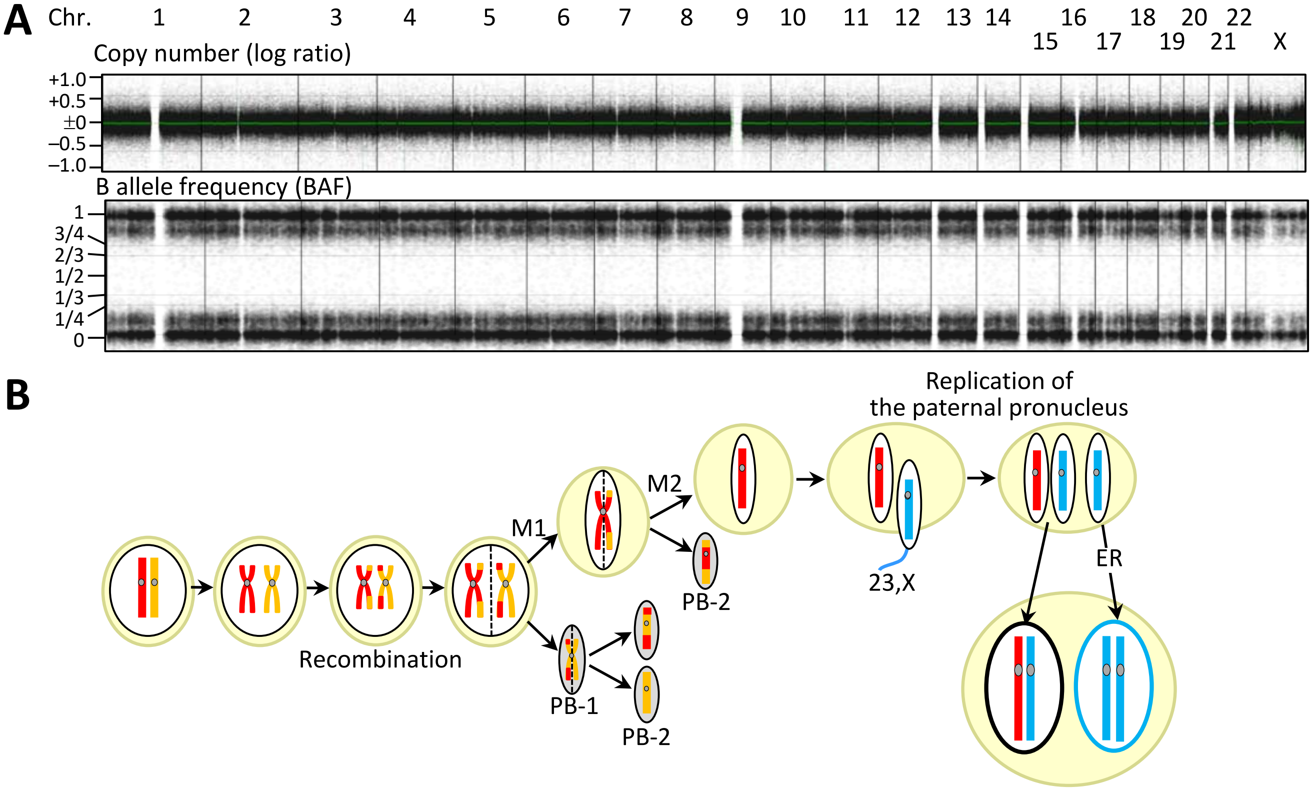

C

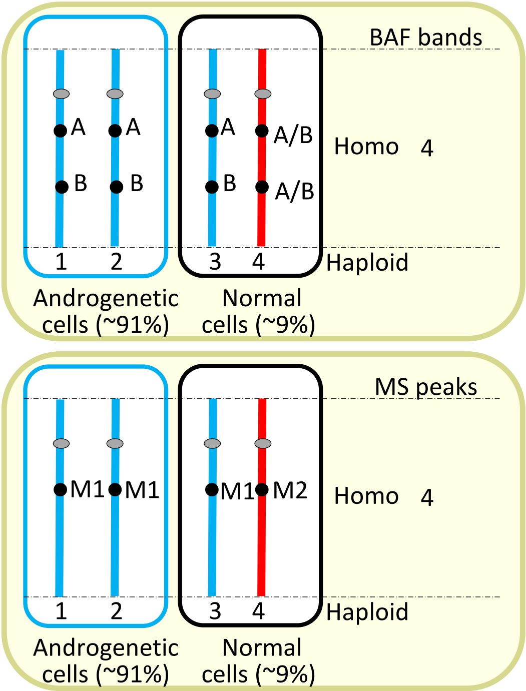

Allele numbers in 100 leukocytes

Haploid 1	Haploid 2	Haploid 3	Haploid 4	BAF
All regions are homozygous between haploids 1–3				
A (91)	A (91)	A (9)	A (9)	0.00 (0/200)
		A (9)	B (9)	0.045 (9/200)
B (91)	B (91)	B (9)	A (9)	0.955 (191/200)
		B (9)	B (9)	1.00 (200/200)

A: reference allele; B: non-refernce allele (B-allele).

Supplementary Figure S4. Generation mechanism of the androgenetic mosaicism in the patient described by Yamazawa et al.

A. Array-based genomewide copy-number and BAF analyses with CytoScan HD, using leukocyte genomic DNA. Copy numbers are apparently normal for all chromosomes, and four BAF bands are identified for all chromosomes.

B. Schematic representation of the generation of the androgenetic mosaicism. For simplicity, the behavior of single homologous chromosomes is shown, and all homologous chromosomes act similarly. It is inferred that, after a normal fertilization between an ovum and a sperm, the paternally derived pronucleus underwent a mitotic division, resulting in the generation of the androgenetic cell lineage by endoreplication of a paternally derived pronucleus and in the formation of the normal cell lineage by union of paternally and maternally derived pronuclei.

C. Evaluation of BAFs and microsatellite (MS) data. It is predicted that the paternally derived two haploid sets in the androgenetic cells and the paternally inherited haploid set in the normal cells are identical. This would produce four BAF bands for all chromosomes. Similarly, this would generate single major MS peaks of paternal origin and single minor MS peaks of maternal origin for all chromosomes when the MS repeat number is different between M1 and M2.

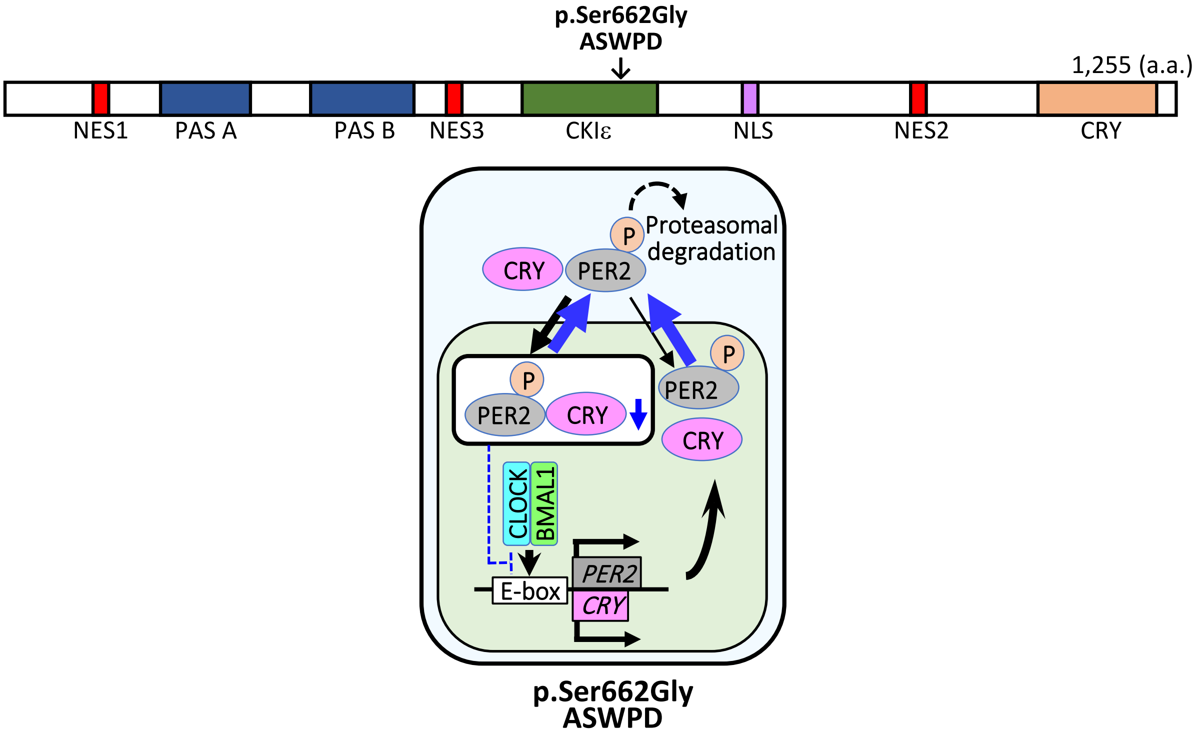

Supplementary Figure S5. The p.Ser662Gly *PER2* variant identified in a dominantly inherited advanced sleep-wake phase disorder (ASWPD) [1]. This variant has been implicated to affect the casein kinase Iε-mediated phosphorylation required for nuclear retention, but not the phosphorylation involved in the proteasomal degradation in the cytoplasm [2–4]. Thus, p.Ser662Gly-PER2 variant would have resulted in premature nuclear export (indicated with thick blue arrows) and earlier cytosolic degradation of PER2-CRY complex, leading to a relative decrease of the PER2-CRY heterodimer in the nucleus (shown with a blue vertical arrow). This would have enhanced PER2 and CRY production (shown with thick black arrows) via a reduced repressing effect of the PER2-CRY dimer on the CLOCK-BMAL1 complex (indicated with thin dotted blue lines), leading to the development of ASWPD.

References

1. Toh KL, Jones CR, He Y, Eide EJ, Hinz WA, Virshup DM, et al. An hPer2 phosphorylation site mutation in familial advanced sleep phase syndrome. Science 2001;291:1040–3.
2. Vanselow K, Vanselow JT, Westermark PO, Reischl S, Maier B, Korte T, et al. Differential effects of PER2 phosphorylation: molecular basis for the human familial advanced sleep phase syndrome (FASPS). Genes Dev 2006;20:2660–72.
3. Albrecht Y, Bordon A, Schmutz I, Ripperger J. The multiple facets of Per2. Cold Spring Harb Symp Quant Biol 2007;72:95–104.
4. Xu Y, Toh KL, Jones CR, Shin JY, Fu YH, Ptácek LJ. Modeling of a human circadian mutation yields insights into clock regulation by PER2. Cell 2007;128:59–70.

SUPPLEMENTARY NOTE

Calculation of the parthenogenetic cell frequency in leukocytes and salivary cells

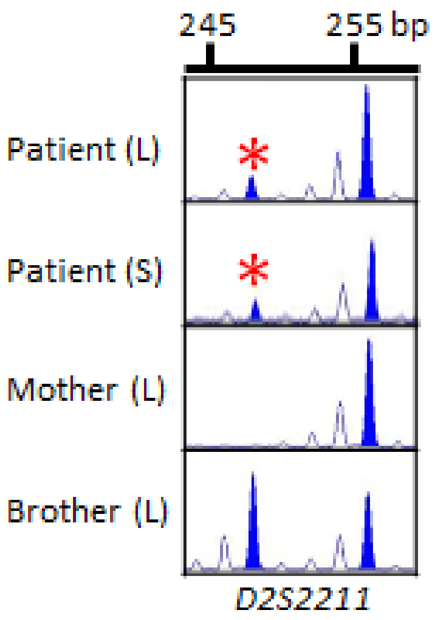

Microsatellite analysis for *D2S2211* has identified two peaks in the brother, and the area under curve (AUC) is larger for the short 248 bp peak than for the long 256 bp peak. This unequal amplification is consistent with short PCR products being more easily amplified than long PCR products. In this patient, the AUC ratio between the minor 248 bp peak of non-maternal (paternal) origin and the major 256 bp peak of maternal origin is obtained as 0.17:1.00 for leukocytes (L) and 0.20:1.00 for salivary cells (S), after compensation of the unequal amplification between the two peaks, using the data in the brother.

Here, let "X" represent the frequency of the parthenogenetic cells in leukocytes (thus, (1 – X) denotes the frequency of normal cells with biparental haploid sets in leukocytes). Then, the non-maternally (paternally) derived 248 bp peak is generated by one paternally derived chromosome in the normal cells, i.e., (1 – X), and the maternally derived 256 bp peak is formed by the products from two maternally derived homologous chromosomes in the parthenogenetic cells and one maternally derived chromosome in the normal 46,XX cells, i.e., (2X + (1 – X)) = (X + 1). Thus, the AUC ratio between the two peaks is represented as (1 – X):(X + 1) = 0.17:1.00, and "X" is obtained as 0.71 (71%). Similarly, when "Y" represents the frequency of the parthenogenetic cells in salivary cells, "Y" is obtained as 0.67 (67%). We performed such calculations for all the informative loci, and the mean frequency is determined as 70% in leukocytes and 67% in salivary cells.
